# Preparation of Naringenin/**β**-Cyclodextrin Complex and Its More Potent Alleviative Effect on Choroidal Neovascularization in Rats

**DOI:** 10.1155/2014/623509

**Published:** 2014-03-27

**Authors:** Xin-rong Xu, Hai-tao Yu, Li Hang, Yan Shao, Shu-hua Ding, Xue-wen Yang

**Affiliations:** ^1^Department of Ophthalmology, Affiliated Hospital of Nanjing University of Traditional Chinese Medicine, 155 Hanzhong Road, Nanjing 210029, China; ^2^College of Pharmacy, Nanjing University of Traditional Chinese Medicine, Nanjing 210023, China; ^3^Department of Clinical Laboratory, Affiliated Hospital of Nanjing University of Traditional Chinese Medicine, Nanjing 210029, China

## Abstract

Choroidal neovascularization (CNV) is characterized by abnormal blood vessels growing from the choroid. Current remedies for CNV have not shown favorable therapeutic efficacy. It is urgent to identify and develop more safe and potent anti-CNV agents via multiple technologies. We previously showed that the natural product naringenin attenuated CNV. However, naringenin has poor water solubility and low bioavailability. Here, we prepared the **β**-cyclodextrin (**β**-CD) complex of naringenin and characterized it using infrared spectra and X-ray diffraction analyses. Determination of content and solubility in the complex showed that naringenin accounted for 20.53% in the complex and its solubility was increased by more than 10-fold. Using a laser-induced CNV model in rats we demonstrated that naringenin/**β**-CD complex more significantly reduced CNV area than naringenin alone in rats. Furthermore, naringenin and its **β**-CD complex significantly inhibited the mRNA and protein expression of VEGF, COX-2, PI3K, p38MAPK, MMP-2, and MMP-9 in retina and choroid tissues. Naringenin/**β**-CD complex showed more significant inhibitory effect on VEGF and COX-2 expression than naringenin. These results collectively indicated that naringenin/**β**-CD complex could be a promising therapeutic option for CNV and that the beneficial effects could be linked to the anti-inflammatory properties of naringenin.

## 1. Introduction

Choroidal neovascularization (CNV) is a common cause of blindness worldwide. It is characterized by new, abnormal blood vessels growing from the choroid through breaks in Bruch's membrane or the basement membrane of the retinal pigment epithelium [1]. These vessels can leak blood and fluid and are accompanied by fibrous tissue, which often leads to damage of the retinal tissues and vision loss. CNV can be classified according to its location in relation to the fovea: subfoveal CNV is located under the center of the retina; juxtafoveal and extrafoveal CNV are located at increasing distances away from the center [2]. CNV is typically secondary to age-related macular degeneration (AMD) and these conditions are major and also substantially increase causes of blindness among aged people.

Several therapeutic options are currently available to treat CNV with variable efficacy on disease progress. Among existing treatments, there are laser photocoagulation, photodynamic therapies, and local corticosteroids. More recently, the use of antiangiogenic remedies has been emerged for CNV treatment. Angiogenic and angiostatic factors have been found to be pivotal in the pathogenesis of CNV. The discovery of such factors as vascular endothelial growth factor (VEGF) and their mechanisms of action has led to the development of drugs specifically targeting the molecules or their signal transduction pathways. VEGF appears to be one of the major regulators in CNV [3, 4]. Consequently, pegaptanib sodium, an anti-VEGF aptamer, was approved for neovascular AMD in 2004 [5], and the anti-VEGF antibody bevacizumab has also been used intravenously [6] and intravitreally [7] to treat CNV.

Although, by these treatments, very effective results are obtained and their further improvement is still possible, it is also reasonable and necessary to look for more successful and definitive alternatives. Currently, natural products isolated from medicinal herbs have been found to be effective remedies for ophthalmologic diseases. Of note, naringenin is a flavanone compound widely found in medicinal plants. Studies have shown that naringenin could significantly prevent the development of CNV induced by laser in rats, which might be related to its effect on choroidal blood flow [8]. However, naringenin has poor water solubility and low bioavailability, which greatly limit its potential utility in the treatment of CNV. The research in this area is already very active and it can be expected that applications of the more recent molecular technologies will bring important advances for CNV remedies. Cyclodextrins are cyclic oligomers of glucose that can form water-soluble inclusion complexes with small molecules and portions of large compounds [9]. These biocompatible, cyclic oligosaccharides do not elicit immune responses and have low toxicities in animals and humans [10]. Of note is that *β*-cyclodextrin (*β*-CD) has been widely used to improve drug delivery and bioavailability [11]. In the current study, we prepared naringenin/*β*-CD complex and characterized its physical and chemical properties, and we further evaluated its efficacy on laser-induced CNV in rats and explored the preliminary mechanisms.

## 2. Materials and Methods

### 2.1. Regents and Antibodies

Naringenin (purity > 98%) and *β*-CD (purity > 98%) were purchased from Beijing Hailin Wei Scientific Co., Ltd. (Beijing, China). The primary antibodies used in western blot analyses against VEGF, COX-2, and p38MAPK were from Cell Signaling Technology (Danvers, MA, USA). The primary antibody against PI3K was from Santa Cruz Biotechnology (Santa Cruz, CA, USA). The primary antibodies against MMP-2, MMP-9, and *β*-actin were from Sigma (St Louis, MO, USA).

### 2.2. Preparation of Naringenin/*β*-CD Complex

Naringenin and *β*-CD (mole ratio 1 : 1) were accurately weighted. Naringenin was first dissolved in a certain amount of ethanol (0.1 mL/10 mg naringenin), and then *β*-CD saturated aqueous solution was slowly added with stirring. The mixture was stirred for 30 min at 60°C and then for additional 5 h without heating. The mixture was cooled naturally and stored overnight at 4°C. The mixture was filtered and the filtrate was dried with freeze dryer for 24 h, and finally the naringenin/*β*-CD complex was obtained. In addition, we also prepared a physical mixture of naringenin and *β*-CD by grinding naringenin and *β*-CD (mole ratio 1 : 1) together in a mortar.

### 2.3. Characterization of Naringenin/*β*-CD Complex

Naringenin/*β*-CD complex of 1.5 mg and KBr of 200 mg were compressed and subjected to infrared spectra analysis (scanning range 4000 cm^−1^ to 400 cm^−1^). The complex samples were also subjected to X-ray diffraction analysis and the detection conditions were as follows: copper target, temperature 50°C, voltage 40 kV, current 100 mA, scanning rate 5°/min, and scanning range 3°–50°.

### 2.4. Determination of Naringenin Concentration in the Prepared Complex

Naringenin of 16.3 mg was dissolved in ethanol and volumed in 100 mL volumetric flask. Solutions of 0.5, 1.0, 1.5, 2.0, 2.5, 3.0, and 3.5 mL were, respectively, volumed with ethanol in 50 mL volumetric flasks. The concentration range was 1.63–11.41 *μ*g/mL. The absorbance (*A*) of these solutions was determined at 288 nm and the standard curve equation was obtained: *A* = 0.0577 × [*C*] + 0.06274 (*r* = 0.9994). Next, a certain amount of naringenin/*β*-CD complex was dissolved in ethanol with the aid of ultrasound and volumed in 100 mL volumetric flask. Solution of 1 mL was volumed with ethanol in 10 mL volumetric flask, and the absorbance was determined at 288 nm. According to the above standard curve equation, the concentration of naringenin in the complex could be calculated.

### 2.5. Determination of Naringenin Solubility in the Prepared Complex

A certain amount of naringenin/*β*-CD complex was dissolved in water to be oversaturated. The solution was shaked within constant temperature air bath at 37°C and 100 r/min for 48 h. The supernatant was filtered with 0.8 *μ*m Millipore filter. The filtrate was diluted to be 90% of the original concentration and its absorbance was determined at 288 nm. According to the above standard curve equation, the solubility of naringenin in the complex could be calculated.

### 2.6. Animal Procedures and Treatments

Male Brown-Norway rats (150–180 g weight) were purchased from Beijing Vital River Experimental Animal Co., Ltd. (Beijing, China). All experimental procedures were approved by the institutional and local committee on the care and use of animals of Nanjing University of Chinese Medicine (Nanjing, China), and all animals received humane care according to the National Institutes of Health (USA) guidelines. Rat CNV model was established according to described methods [12]. Briefly, rats were anesthetized using intramuscular injection with ketamine (50 mg/kg) and then a mixture of 0.5% tropicamide and phenylephrine was used for mydriasis. Krypton laser was concentrated to eight spots between the large retinal vessels at an optic disk. The parameters of laser radiation were 568 nm wavelength, 100 *μ*m spot diameter, 0.1 s exposure duration, and 150–200 mW power. The spots where tiny bubbles appeared after the Bruch membrane was broken were viewed to be qualified. Hemorrhage of retina, choroid, or vitreum was excluded in CNV model establishment. Twenty-four CNV rats were randomly divided into three groups (eight rats/each group), namely, model group, naringenin group, and naringenin/*β*-CD complex group. Treatments began following retinal photocoagulation and rats in treatment groups were intraperitoneally injected with naringenin (20 mg/kg, dissolved in DMSO) or naringenin/*β*-CD complex (20 mg/kg) once daily for four weeks. Rats in model group were intraperitoneally injected with DMSO of equal volume. In addition, eight rats were normally raised without any manipulation as control group.

### 2.7. Measurement of CNV Area

After four-week treatment, rats (3 rats (6 eyes)/group) were subjected to sublingual injection with 10% FITC-D (0.2 mL/rat). One hour later, the eyeballs were removed and fixed in 40 g/L paraformaldehyde. The eyeballs were opened along the equator of eyeball under a microscope. Anterior segments and retinal nerve fibre layer were removed, and the retinal pigment epithelium-choroid-sclera complexes were obtained. With optic nerve as a center, the complex was cut open radially. The retinal pigment epithelium was inverted on cover glass and the graphs were taken using a fluorescence microscope (magnification ×40). ImageJ Software was used to quantify the graphs.

### 2.8. Real-Time PCR

Total RNA was isolated from retina and choroid of rats (2 rats (4 eyes)/group) using Trizol reagent (Sigma, St Louis, MO, USA) following the protocol provided by the manufacturer. Real-time PCR was performed as described previously [13]. *β*-Actin was used as the invariant control. Fold changes in the mRNA levels of target genes related to the invariant control glyceraldehyde phosphate dehydrogenase (GAPDH) were calculated as suggested by Schmittgen et al. [14]. The following primers were used in real-time PCR analyses: COX-2 (forward) 5′-TACGAAGACCCTGCCTACGA-3′, (reverse) 5′-GTTGGTGGCAAGTGAAGCTG-3′; VEGF (forward) 5′-CTTGCAGATGTGACAAGCCAAG-3′, (reverse) 5′-GGTGTGGTGGTGACATGGTTA-3′; PI3K (forward) 5′-CAGCACCTCGTATGGCTCAAT-3′, (reverse) 5′-GTCCTCACTCTTGATCCCCAG-3′; p38MAPK (forward) 5′-GTTGTCCTCCCTCCTCGTTC-3′, (reverse) 5′-GTTACCGCTCGACTTGTGCT-3′; MMP-2 (forward) 5′-CCGTTATGAGACCCTGAGCC-3′, (reverse) 5′-CAGACCAATCGTGCCTCCAT-3′; MMP-9 (forward) 5′-CGGATCCCCCAACCTTTACC-3′, (reverse) 5′-AGCCAGCTGAGTTCAATCCC-3′; *β*-actin (forward) 5′-CCCATCTATGAGGGTTACGC-3′, (reverse) 5′-TTTAATGTCACGCACGATTTC-3′.

### 2.9. Western Blot Analyses

The retina and choroid tissues of rats (3 rats (6 eyes)/group) were homogenised in RIPA lysis buffer (0.1% SDS, 0.5% deoxycholate, 1% Nonidet, 150 mM NaCl, and 50 mM Tris-HCl) containing protease inhibitors on ice. The protein levels were determined using a BCA assay kit (Pierce, USA). Proteins (50 *μ*g/well) were separated by SDS-polyacrylamide gel, transferred to a PVDF membrane (Millipore, Burlington, MA, USA), blocked with 5% skim milk in Tris-buffered saline containing 0.1% Tween 20. Target proteins were detected by corresponding primary antibodies and subsequently by horseradish peroxidase-conjugated secondary antibodies. Protein bands were visualized using chemiluminescence reagent (Millipore, Burlington, MA, USA). Equivalent loading was confirmed using an antibody against *β*-actin. The levels of target protein bands were densitometrically determined using Quantity One 4.4.1. The variation in the density of bands was expressed as fold changes compared to the control in the blot after normalization to *β*-actin.

### 2.10. Statistical Analysis

Data were presented as mean ± SD, and results were analyzed using SPSS16.0 software. The significance of difference was determined by one-way ANOVA with the post hoc Dunnett's test. A value of *P* < 0.05 was considered to be statistically significant.

## 3. Results

### 3.1. Characterization and Properties of Naringenin/*β*-CD Complex

**Figure 1:**
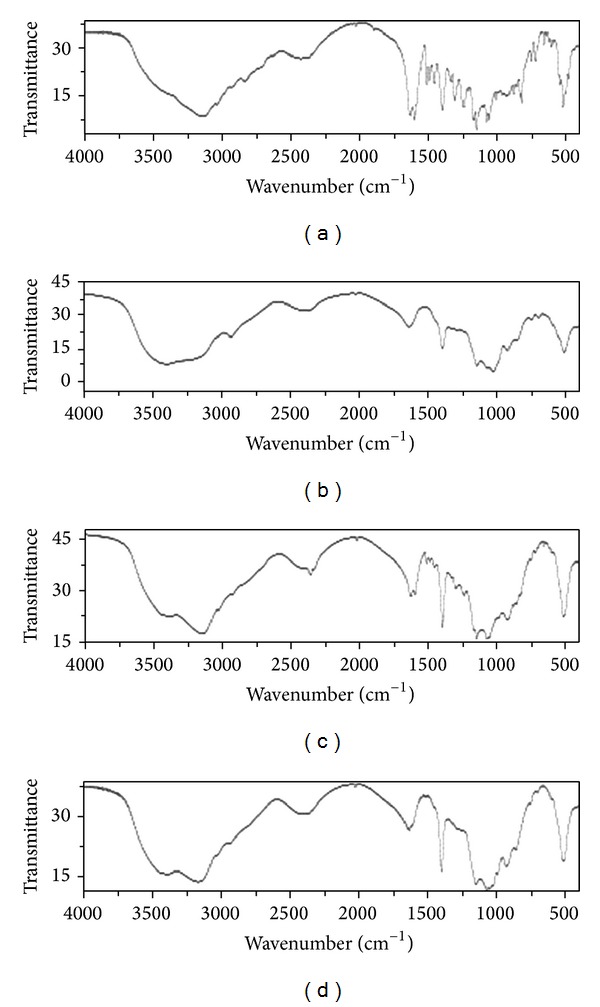
Infrared spectra analyses of *β*-CD (a), naringenin (b), physical mixture of naringenin and *β*-CD (c), and naringenin/*β*-CD complex (d).

**Figure 2:**
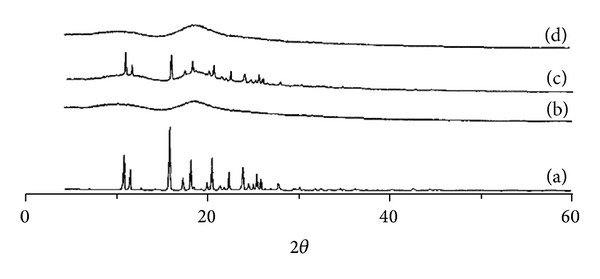
X-ray diffraction analyses of *β*-CD (a), naringenin (b), physical mixture of naringenin and *β*-CD (c), and naringenin/*β*-CD complex (d).

Infrared spectrum analyses showed that naringenin had strong absorption at the wave number ranges of 1790–1540, 1530–1200, and 960–750 cm^−1^. In naringenin/*β*-CD complex, red shift occurred in naringenin absorption due to *β*-CD inclusion. However, the infrared spectrum of physical mixture of naringenin and *β*-CD was obviously a superposition of their individual spectrums (Figure 1). In X-ray diffraction analysis, naringenin had a series of strong diffraction peaks at the positions of 10.7°, 11.4°, 15.6°, 17.3°, 15.2°, 20.6°, 22.3°, 23.7°, 25.7°, and 27.8°, indicating its crystal property. However, *β*-CD showed several wide peaks suggesting its amorphous property. The physical mixture of naringenin and *β*-CD showed superimposed diffraction peaks of naringenin and *β*-CD without new peaks, but the peak intensity had some changes. Naringenin/*β*-CD complex showed two wide diffraction peaks of *β*-CD and the characteristic peaks of naringenin disappeared (Figure 2). All these spectrum analyses above indicated that naringenin was included in *β*-CD and the naringenin/*β*-CD complex was successfully prepared. Subsequently, we examined some physical properties of naringenin/*β*-CD complex. Our results demonstrated that the inclusion rate was 81.06% and that the content of naringenin in the complex reached 20.53%. In addition, the water solubility of naringenin in the complex was enhanced more than 10-fold compared to that of pure naringenin (Table 1), suggesting that naringenin/*β*-CD complex could have better bioavailability and bioactivity.

### 3.2. Naringenin/*β*-CD Complex Significantly Reduces CNV Area in Rats

**Figure 3:**
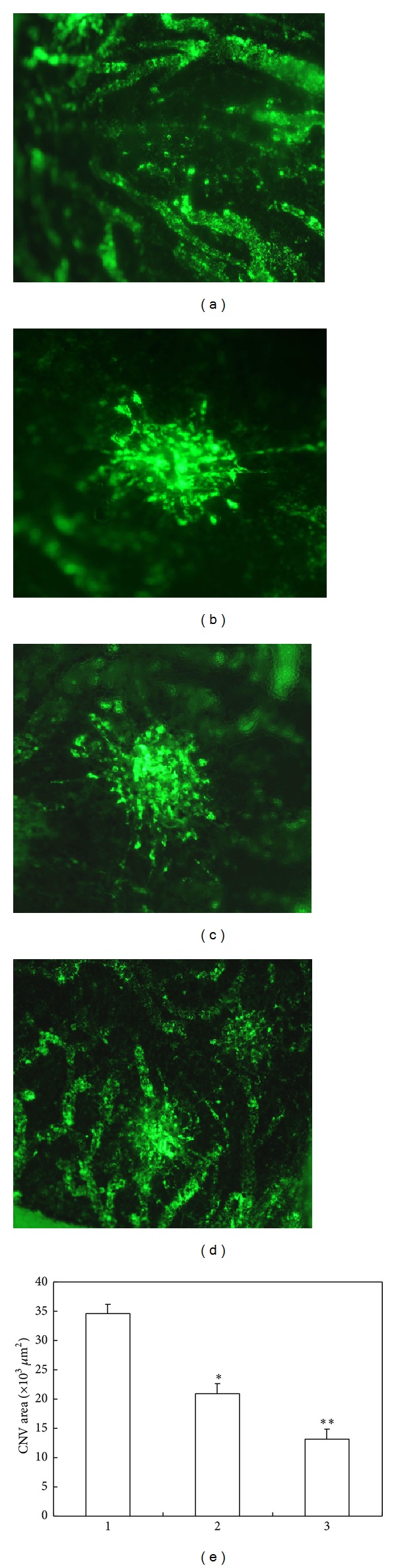
Naringenin/*β*-CD complex significantly reduces CNV area in rats. (a)–(d) are representative fluorescent graphs of choroidal flat mounts with FITC-D in the four groups. (e) is the quantification of CNV area in rats. Group 1: CNV rats without treatment; group 2: CNV rats with naringenin treatment; group 3: CNV rats with naringenin/*β*-CD complex treatment. Data are expressed as mean ± SD; ^#^ *P* < 0.05 versus group 1, *P < 0.05 versus group 1, and **P < 0.01 versus group 1.

Examination of CNV area showed that the rats in model group had considerably larger CNV area compared to the normal rats. However, the CNV area in treatment rats was significantly reduced by naringenin (*P* < 0.05) or naringenin/*β*-CD complex (*P* < 0.01) compared to the control rats. In addition, the CNV area in rats treated with naringenin/*β*-CD complex was also significantly lower than that of rats treated with naringenin (*P* < 0.05) (Figure 3). These data indicated that naringenin could effectively attenuate CNV in rats and that the naringenin/*β*-CD complex had more potent effect.

### 3.3. Naringenin/*β*-CD Complex Significantly Inhibits the Expression of Key Mediators Involved in the Pathogenesis of CNV in Rats

**Figure 4:**
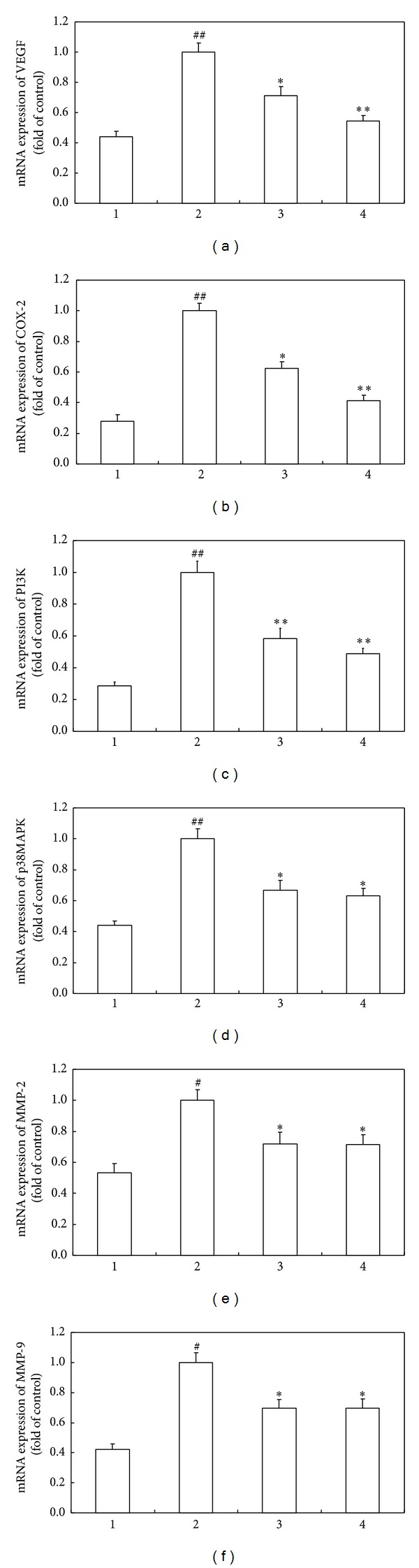
Naringenin/*β*-CD complex significantly inhibits the mRNA expression of key mediators involved in the pathogenesis of CNV in rats. (a)–(f) are real-time PCR analyses of transcript levels of VEGF, COX-2, PI3K, p38MAPK, MMP-2, and MMP-9. GAPDH was used as the invariant control for calculating fold changes in mRNA levels. Group 1: normal rats; group 2: CNV rats without treatment; group 3: CNV rats with naringenin treatment; group 4: CNV rats with naringenin/*β*-CD complex treatment. Data are expressed as mean ± SD; ^#^ *P* < 0.05 versus group 1, ^##^ *P* < 0.01 versus group 1, *P < 0.05 versus group 2, and **P < 0.01 versus group 2.

**Figure 5:**
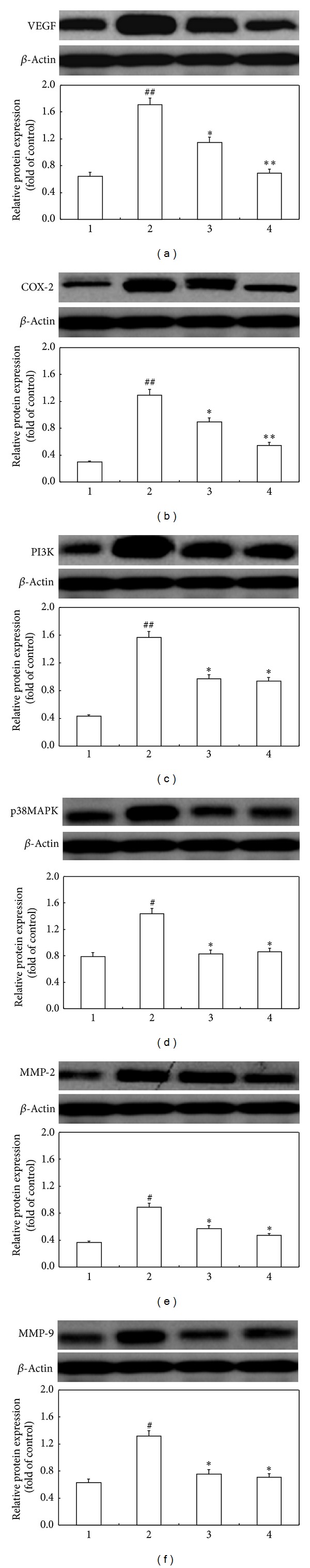
Naringenin/*β*-CD complex significantly inhibits the protein expression of key mediators involved in the pathogenesis of CNV in rats. (a)–(f) are western blot analyses for protein levels of VEGF, COX-2, PI3K, p38MAPK, MMP-2, and MMP-9 and their quantification. *β*-Actin was used as an invariant control for equal loading. Group 1: normal rats; group 2: CNV rats without treatment; group 3: CNV rats with naringenin treatment; group 4: CNV rats with naringenin/*β*-CD complex treatment. Data are expressed as mean ± SD; ^#^ *P* < 0.05 versus group 1, ^##^ *P* < 0.01 versus group 1, *P < 0.05 versus group 2, and **P < 0.01 versus group 2.

A number of mediators have been demonstrated to be involved in the pathogenesis of CNV [1]. We subsequently examined the expression of VEGF, cyclooxygenase-2 (COX-2), phosphatidylinositol-3-kinase (PI3K), p38 mitogen-activated protein kinase (p38MAPK), matrix metalloproteinase (MMP)-2, and MMP-9 in retina and choroid tissues of rats. Real-time PCR analyses showed that the expression of the above molecules was significantly upregulated in the CNV rats without treatment. However, both naringenin and naringenin/*β*-CD complex considerably reduced their mRNA expression to different extents. Of note, naringenin/*β*-CD complex produced more potent reducing effects on the gene expression of VEGF and COX-2 (Figure 4). We also examined their protein expression of these molecules using western blot assays, showing that both naringenin and naringenin/*β*-CD complex significantly diminished the increased expression of these molecules at protein level. Similarly, the protein abundance of VEGF and COX-2 was significantly reduced by naringenin/*β*-CD complex compared to that by single naringenin treatment (Figure 5). Taken together, these results suggested that naringenin and its *β*-CD complex could downregulate the expression of some key mediators in retina and choroid tissues in rats.

## 4. Discussion

Classic CNV are associated with bright, early hyperfluorescence that is often well defined and that leaks in the later phase of the angiogram [15]. CNV is an important cause of vision loss in younger patients. Untreated CNV can cause rapid deterioration of central vision and is associated with a poor prognosis. Until recently, many patients with subfoveal CNV could not be treated because of the risk of central vision loss associated with laser photocoagulation [16]. The knowledge of molecular factors and processes involved in a specific pathology represents now one of the major starting points for the identification of new drugs.

Natural compounds have been usually the source for novel drug screening and discovery. Naringenin can be widely found in grape juice, lemon, orange, and Poncirustrifoliate and has a wide range of bioactivities including anti-inflammatory, antioxidant, antiatherosclerotic, and anticancer effects [17]. Previous study showed that naringenin could improve the choroidal blood flow and inhibit the expression of COX-2 and iNOS, leading to downregulation of VEGF expression and inhibition of CNV formation [18]. However, naringenin suffers from poor water solubility and low bioavailability. Current technologies in pharmaceutics material sciences allow for the production of target specific and/or function-specific chemical ligands and drugs [19]. Cyclodextrin structurally contains an electron-rich hydrophobic cavity and hydrophilic outer wall [20]. Cyclodextrin has been commonly used as a carrier for improving the deficiency of drug molecules, especially those who are water-insoluble and unstable. For example, Zhang and Cui prepared P-glycoprotein substrate berberine hydrochloride/HP-*β*-CD complex and used single-pass intestinal perfusion method to evaluate the effect of cyclodextrin infusion on the intestinal absorption of berberine hydrochloride in rats, and they found that HP-*β*-CD inclusion significantly enhanced the absorption rate constant and the permeability coefficient of berberine hydrochloride in small intestine [21]. Shulman and Cohen demonstrated that HP-*β*-CD inclusion increased the water solubility of naringenin by 400-fold and enhanced the transport capacity crossing Caco-2 cells by 11-fold. Further, their pharmacokinetic data showed that AUC was increased by 7.4-fold and *C*_max⁡_ increased by 14.6-fold in rats [22]. In the present study, we prepared the naringenin/*β*-CD complex and characterized it using X-ray diffraction and FTIR methods. Determination of solubility showed 11.8-fold increases in water solubility of naringenin. Using laser-induced CNV model in rats, we further demonstrated that naringenin/*β*-CD complex had more potent inhibitory effects on CNV than those of naringenin alone, suggesting the significant improvement of bioavailability and biological activity by *β*-CD inclusion.

Studies have shown that inflammation plays an important role in the formation of CNV [23]. COX-2 is a critical regulatory enzyme in inflammatory processes and is closely associated with the formation of ocular neovascularization [24]. COX-2 can promote angiogenesis via pathways mediated by prostaglandins, thromboxane A2, and MMPs, of which VEGF-MMP system plays an important role in endothelial cell proliferation and migration and the eventual formation of new blood vessels [25, 26]. The CNV model was induced by laser in the current study, and it was actually manifestation of the inflammatory responses induced by thermal injury. Our data showed that the mRNA and protein expression of VEGF, MMP-2, and MMP-9 were significantly upregulated in CNV rats, confirming the critical role of VEGF-MMP system in the formation of CNV. Meanwhile, the expression of COX-2 was also increased concomitant with upregulation of VEGF and MMPs, suggesting that laser-induced CNV could be associated with COX-2 induction of VEGF and MMPs. We found that naringin and naringin/*β*-CD complex could downregulate the expression of these molecules and inhibit CNV formation, presumably due to the anti-inflammatory effect of naringin resulting in the downregulated expression of COX-2 as well as VEGF-MMP system. Our results also showed that naringin/*β*-CD complex, compared to naringin alone, had more effective inhibitory effect on the expression of COX-2 and VEGF, although all the tested molecules could be downregulated by naringin and its *β*-CD complex. It could be postulated that naringin could strongly inhibit the production of prostaglandin E2 resulting in potent suppression of COX-2 and VEGF.

The present study also investigated the role of PI3K and MAPK signal pathways in the formation of CNV. These two cascades can be activated by inflammation and injury and regulate cell proliferation, differentiation, apoptosis, and motility, and they are also involved in COX-2 activity [27, 28]. PI3K is a type of kinase specifically catalyzing the 3-hydroxyl phosphorylation of phosphatidylinositol, which generates inositol lipids as second messengers such as p70S6K, protein kinase B and AKT. The PI3K/AKT pathway is a primary downstream signal transduction of PI3K. COX-2 can activate the serine-threonine kinase, leading to activation of AKT signal pathway, while celecoxib could dose-dependently inhibit AKT phosphorylation in vascular endothelial progenitor cells and cause apoptosis [29]. MAPK pathways are important signal transduction systems within cells, of which p38MAPK is commonly activated by inflammatory factors, stress stimulation, lipopolysaccharide, protein synthesis inhibitor, and bacterial pathogens [30]. Activated p38 translocates to the nucleus and transcriptionally regulates the expression of genes involved in immune modulation, inflammatory responses, and apoptosis under stress [30]. COX-2 is localized in endoplasmic reticulum and nuclear membrane and catalytically produces prostaglandin E2 within cells increasing activities of MAPKs [31]. Studies showed that p38 pathway activation could enhance interleukin-1*β*-induced COX expression and increase prostaglandin E2 synthesis, but these effects could be completely abrogated by p38 inhibitor SC68376 [32]. Our current study revealed that PI3K and p38MAPK expression was significantly upregulated concomitant with upregulation of COX-2, VEGF, MMP-2, and MMP-9 in CNV animals, indicating that PI3K and p38MAPK signal systems were related to the formation of CNV. Naringenin and its *β*-CD complex could downregulate the expression of PI3K and p38MAPK, possibly due to the inhibition of COX-2. This could also be supported by the observation that significant differences only existed in the expression of COX-2 and VEGF, rather than other tested molecules, altered by naringenin and its *β*-CD complex. Further investigations are needed to explore whether some other pathways mediated naringenin regulation of PI3K and p38MAPK or naringenin directly regulated them.

In summary, the prepared naringenin/*β*-CD complex showed increased water solubility and improved biological activity leading to more potent inhibitory effect on CNV formation in rats. PI3K and p38MAPK signal pathways were involved in the pathogenesis of CNV. Attenuation of CNV by naringenin and its *β*-CD complex could be associated with the anti-inflammatory properties especially with the inhibition of COX-2.

## Figures and Tables

**Table 1:** Several properties of naringenin and its *β*-CD complex.

	Inclusion rate	Naringenin content	Naringenin solubility (*μ*g/mL)
Naringenin	N/A	N/A	71.85
Naringenin/*β*-CD complex	81.06%	20.53%	846.28

Note: N/A means not applicable.
